# Banff 2016 Global Assessment and Quantitative Scoring for T Cell-Mediated Liver Transplant Rejection are Interchangeable

**DOI:** 10.1155/2023/3103335

**Published:** 2023-03-27

**Authors:** Maryam Eghtedari, Catriona McKenzie, Lauren C. Y. Tang, Avik Majumdar, James G. Kench

**Affiliations:** ^1^Central Clinical School, University of Sydney, Camperdown, NSW, Australia; ^2^Royal Prince Alfred Hospital, Camperdown, NSW, Australia; ^3^Department of Tissue Pathology and Diagnostic Oncology, NSW Health Pathology, Royal Prince Alfred Hospital, Camperdown, NSW, Australia; ^4^AW Morrow Gastroenterology and Liver Centre, Royal Prince Alfred Hospital, Camperdown, NSW, Australia

## Abstract

**Introduction:**

Histopathological assessment of liver biopsies is the current “gold standard” for diagnosing graft dysfunction after liver transplantation (LT), as graft dysfunction can have nonspecific clinical presentations and inconsistent patterns of liver biochemical dysfunction. Most commonly, post-LT, graft dysfunction within the first year, is due to acute T-cell mediated rejection (TCMR) which is characterised histologically by the degree of portal inflammation (PI), bile duct damage (BDD), and venous endothelial inflammation (VEI). This study aimed to establish the relationship between global assessment, which is the global grading of rejection using a “gestalt” approach, and the rejection activity index (RAI) of each component of TCMR as described in revised Banff 2016 guidelines.

**Methods:**

Liver biopsies (*n* = 90) taken from patients who underwent LT in 2015 and 2016 at the Australian National Liver Transplant Unit were identified from the electronic medical records. All biopsy slides were microscopically graded by at least two assessors independently using the revised 2016 Banff criteria. Data were analysed using IBM SPSS v21. A Fisher–Freeman–Halton test was performed to assess the correlation between the global assessment and the RAI scores for each TCMR biopsy.

**Results:**

Within the cohort, 60 (37%, *n* = 164) patients underwent at least 1 biopsy within 12 months after LT. The most common biopsy outcome (total *n* = 90) was acute TCMR (64, 71.1%). Global assessment of TCMR slides strongly positively correlated with PI (*p* value <0.001), BDD (*p* value <0.001), VEI (*p* value <0.001), and total RAI (*p* value <0.001). Liver biochemistry of patients with TCMR significantly improved within 4 to 6 weeks post-biopsy compared to the day of the biopsy.

**Conclusion:**

In acute TCMR, global assessment and total RAI are strongly correlated and can be used interchangeably to describe the severity of TCMR.

## 1. Introduction

Liver transplant (LT) is a potentially life-saving procedure for advanced-stage liver diseases [1] with the most common indications for LT in Australia and New Zealand being hepatocellular carcinoma (HCC), alcohol-related cirrhosis, and nonalcoholic fatty liver disease (NAFLD) [2, 3].

Liver biopsy is the “gold standard” tool to diagnose liver allograft rejection in the setting of transplantation [4]. Rejection is a serious cause of liver allograft dysfunction, which can lead to adverse outcomes such as graft loss [5, 6]. Risk factors for graft rejection include fewer human leukocyte antigen matches between the donor and the recipient, noncompliance with treatment, increased graft cold ischaemic time (CIT), and autoimmune disease as an indication for transplant [5, 7, 8]. Clinically, liver rejection presents with nonspecific findings including elevated liver biochemistry. These findings have a wide range of differential diagnoses and are shared by other causes of graft dysfunction including ischaemia-reperfusion injury, recurrence of original liver disease, and biliary complications [9]. Therefore, histopathological assessment of liver biopsies is currently the most reliable method for diagnosing graft rejection. These biopsies are most commonly performed percutaneously and are not without risk, with serious complication and mortality rates reported to be 1% and 0.2%, respectively [10, 11]. Hence, it is important to ensure that histological assessment and associated scoring systems provide reliable and consistent data to inform clinical management decisions.

In 1997, the Banff Working Group, comprising experts in liver transplant pathology, hepatology, and surgery, developed and published an international consensus framework for the grading of acute liver allograft rejection. They proposed both a global grading of rejection using a “gestalt” approach, and a semiquantitative system assigning numerical scores to different histological parameters [12]. This numerical grading system, known as the rejection activity index (RAI), is based on scoring features in 3 categories: portal inflammation (PI), bile duct inflammation damage (BDD), and venous endothelial inflammation (VEI). The limited data published on the concordance between these two systems have yielded somewhat divergent results [12, 13]. In the 2016 Comprehensive Update of the Banff Working Group on Liver Allograft Pathology, the criteria for both global assessment (GA) and RAI quantitative scoring were revised, in particular to take into account the presence of perivenular inflammation and necrosis in some cases (Supp Table 1) [14–16]. Given the divergent findings on the concordance between global grade and RAI in previous reports using the 1997 Banff criteria and the lack of data on this issue following the 2016 Banff revisions, we have investigated the relationship between Banff 2016 global assessment and RAI severity scores in a histologically well characterised cohort from the Australian National Liver Transplant Unit (ANLTU) at Royal Prince Alfred Hospital (RPAH).

## 2. Materials and Methods

Ethics approval for this study was obtained from the Sydney Local Health District (SLHD) Human Ethics Review Committee (Protocol No. X16-0493), and retrospective clinical data were collected from Royal RPAH Pathology Department, ANLTU databases, and the electronic medical records. The patient cohort included each consecutive adult (age ≥18 years) patient who underwent LT at RPAH in 2015 and 2016. All patients meeting these criteria were included as part of the patient cohort with no other inclusion or exclusion criteria. All patients from this cohort who had liver biopsies performed within 12 months following LT in 2015 and 2016 were identified and were analysed as part of a biopsy subgroup with no other inclusion or exclusion criteria.

Each biopsy was reviewed to confirm the original diagnosis, and those with a diagnosis of TCMR were graded independently according to the 2016 Banff criteria (Demetris et al. 2016) by two assessors: a senior pathologist (CM) with expertise in liver transplantation pathology and a junior doctor (ME). Discrepancies were resolved by the addition of a third assessor, also a senior pathologist with expertise in liver transplantation pathology (JGK), and multiheader microscope consensus conferencing. Both global assessment grade and quantitative RAI scores for each category and overall were recorded. Total RAI scores were grouped 1-2 = indeterminate, 3-5 = mild, 6-7 = moderate, and 8-9 = severe (slightly modified from original groupings proposed in Banff 1997 by adding score 3, “consistent with” to the mild group) [12].

Laboratory data collected included alanine aminotransferase (ALT), gamma-glutamyl transpeptidase (GGT), and alkaline phosphatase (ALP) which were collected on the day of the biopsy and were followed up at 4 to 6 weeks postbiopsy; this time frame is chosen to account for the potential normalisation of the liver biochemistry and effect of response to treatment. The patients who received pulse intravenous (IV) methylprednisolone following a biopsy TCMR diagnosis were identified from the medical record.

## 3. Statistical Analysis

Quantitative data are expressed as frequency tables. A paired *t*-test was performed to compare the liver biochemistry values at 4 to 6 weeks after the biopsy compared to the day of the biopsy. A Fisher–Freeman–Halton test was performed to assess the correlation between the global assessment and the RAI scores for each TCMR biopsy. In order to assess correlation, mild, moderate, and severe categories of global assessment were given scores of 1, 2, and 3, respectively. Statistical analyses were conducted using IBM SPSS Statistics v21, and all statistical analyses were 2-tailed with the significance level set at 0.05.

## 4. Results

This study identified 164 patients who underwent LT at RPAH in 2015 and 2016, and the characteristics of the patient cohort are included in Table 1. The most common primary indication for LT was hepatitis C cirrhosis (39, 24%) (Table 2). More than a third (60, 37%) of these patients (*n* = 164) underwent at least one liver biopsy within 12 months post-LT. In 60% of the cases, the biopsy was performed within 90 days from the date of LT, and the most common indications for performing a biopsy were clinical suspicion of rejection. The most frequent biopsy diagnosis in our study was TCMR (71.1%, *n* = 90) (Table 3). The most common indications for transplantation in the population of patients who experienced TCMR were also hepatitis C (22%) and alcohol-related cirrhosis (22%). LT recipients who had TCMR as a complication had a CIT of 6.86 hours compared to an average CIT of 6.41 for the entire cohort [5]. No antibody-mediated rejection was identified in our cohort. Patients received protocol induction immunosuppression with either methylprednisolone, methylprednisolone plus basiliximab, or a steroid-free protocol with basiliximab alone in selected NAFLD patients or those with HCV viraemia. Maintenance immunosuppression regimens were tacrolimus or cyclosporin based, with selective use of antiproliferative agents (mycophenolate or azathioprine) and maintenance prednisolone as per local unit protocol. Sirolimus or everolimus was used on a case-by-case basis.

**Figure 1:**
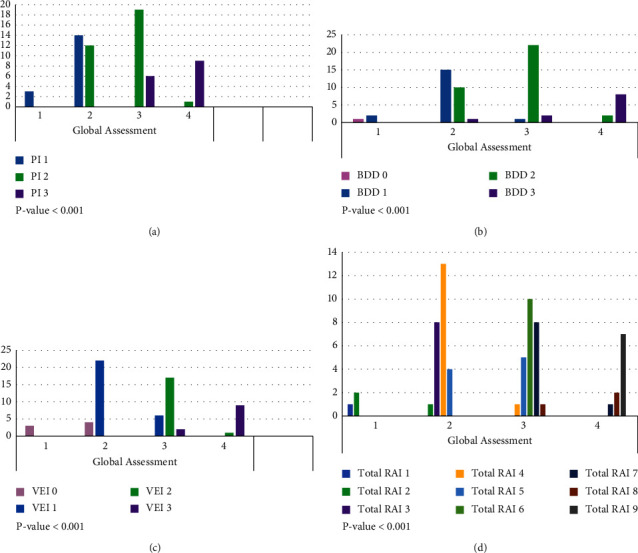
Clustered bar count of global assessment by (a) portal inflammation (PI); (b) bile duct damage (BDD); (c) venous endothelial inflammation (VEI); and (d) total rejection activity index (RAI) categories for T-cell mediated rejection biopsy. *P* values indicate the Fisher–Freeman–Halton correlations between global assessment and PI, BDD, VEI, and total score. Global assessment values: 1 corresponds to the intermediate category; 2 to mild; 3 to moderate; and 4 to severe.

The proportions of mild, moderate, and severe TCMR (total *n* = 90) were 40.6%, 39%, and 15.6%, respectively, and the RAI groups 3–5, 6-7, and 8-9 comprised 48.4%, 29.7%, and 15.6%, respectively (Table 4) [14]. Amongst the 64 biopsies with TCMR, 10 (15.6%) were associated with a different severity category group when scored using RAI vs. global assessment. In 8 of the 9 cases, the severity group was downgraded in RAI compared to the global assessment. The breakdown of the change in category (from GA to RAI assessment) included mild to no rejection/indeterminate (*n* = 1, 11%), moderate to mild (*n* = 6, 67%), severe to moderate (*n* = 1, 11%), and moderate to severe (*n* = 1, 11%). In two cases where the severity was downgraded to mild (RAI) from moderate (GA) grading, the patients were pulsed with methylprednisolone. In biopsies with TCMR, global assessment is positively correlated with PI (*p* value < 0.001), BDD (*p* value <0.001), VEI (*p* value <0.001), and total RAI (*p* value <0.001) scores (Figure 1).

Liver biochemistry values at 4 to 6 weeks after the biopsies compared to the day of the biopsy were analysed, and these values had reduced significantly for ALT, ALP, and GGT at the latter time point. The biopsies with the global assessment of severe and moderate had statistically significant increased mean ALT, ALP, and GGT levels compared to the biopsies with the global assessment of mild and indeterminate. In 42% (*n* = 27, total = 60) of the cases of TCMR, patients received IV methylprednisolone as an inpatient following biopsy; of these, 74% (*n* = 20) had either moderate or severe rejection per global assessment criteria. In the cases of mild TCMR who received methylprednisolone IV pulse (*n* = 7), 2 (29%) cases had multiple biopsies, and subsequent biopsies showed severe TCMR and resolving TCMR, respectively.

## 5. Discussion

The Banff schema for grading liver allograft rejection has been widely adopted and is regarded as a useful index of the severity of TCMR [13, 17–20]. The system was originally published in 1997 and updated in 2016; however, there are limited data on whether there are differences between global grading and RAI. Hence, the possibility that global assessment may underestimate or overestimate the severity of rejection based on a semiquantitative analysis, an issue raised in the original description of the Banff schema, cannot be dismissed [12]. We have demonstrated for the first time using the contemporary Banff 2016 definitions that there is an excellent correlation between the two methods, and that either can be used to guide clinical care. Not only was there strong correlation between the total RAI score and global grade assessment, there was also a significant correlation between the score for each individual category and global grade, particularly for venous endothelial inflammation.

In the 1997 Banff International Consensus Document, the lead author notes unpublished data that evaluation of a series of 50 post-transplantation liver allograft biopsy specimens by himself using both methods showed no significant differences between the two systems. [12] Höroldt et al. regarded the two methods as “not interchangeable,” giving an example of a biopsy where the overall global assessment is moderate while the total RAI score would be classified as severe [13]. However, in their analysis of 231 patients diagnosed with acute cellular rejection, the two Banff 1997 methods of grading rejection showed good agreement with each other (kappa 0.70). In 2016, the Banff schemas for grading TCMR were upgraded with changes to the criteria for both global assessment and quantitative scoring, and though the changes could potentially result in cases being assigned different grades, we have shown that there is still a significant correlation between the two systems and that either is suitable for assessing the severity of rejection.

The biopsy rate seen in our centre (37%, *n* = 164) was similar to that reported in a multicentre study in the UK and Spain (30%, *n* = 470) [21] and like us, a study from Germany also reported TCMR as the most common outcome of liver biopsy (36%, *n* = 496), although no data on the severity of rejection changes were reported in the latter study [22]. Identifying the rate of moderate to severe rejection is important as this group of patients is more likely to require admission LT for pulsed steroids and increased baseline immunosuppression. The rate of moderate to severe rejection in our study was 54% (*n* = 35) based on global assessment and 45% (*n* = 29) based on RAI score groupings, both of which are higher than the 34% moderate/severe rate (*n* = 142) reported by Rodríguez–Perálvarez et al. [21], possibly reflecting differences in patient population, immunosuppression, and biopsy protocols. In our study, 74% of patients who received IV methylprednisolone as an inpatient had either moderate or severe TCMR. Both the improvement in liver biochemistry and administration of IV methylprednisolone following biopsy suggest that information from liver biopsies plays an important role in the management of liver rejection post-LT. However, we cannot draw any firm conclusion since; although we were able to obtain robust histopathological data, the retrospective nature of our study led to limitations in the collection of clinical data, including changes in liver biochemistry and treatments before and after the biopsies.

## 6. Conclusion

TCMR was the most common finding in post-transplant liver biopsies. The 2016 Banff criteria for global assessment and total RAI can potentially be used interchangeably to assess the severity of the rejection episode and help guide clinical management.

## Figures and Tables

**Table 1:** Details of the patients who underwent liver transplantation at Royal Prince Alfred Hospital in 2015 and 16 and patients from the cohort who were diagnosed with biopsy-proven T-cell mediated rejection (TCMR) within one year of transplantation.

Demographics	Patients who underwent liver transplantation at Royal Prince Alfred Hospital in 2015 and 16	Patients with TCMR
Number of liver transplantations in 2015 and 16	164	51
Donor: DBD or DCD^∗^	DBD: 154 (94%, *n* = 164) and DCD: 10 (6%, *n* = 164)	DBD: 46 (90%, *n* = 51) and DCD: 5 (6%, *n* = 51)
Average CIT ^∗^ (hours)	6.41 (*s* = 2.14)	6.86 (*s* = 2.03)
Sex	Male 117 (71%, *n* = 164) female 47 (29%, *n* = 164)	Male 35 (67%, *n* = 51) female 16 (33%, *n* = 51)
Average age at transplant	53.8 years	53.0 years

^∗^Donation after brain death (DBD) and donation after cardiac death (DCD). ^∗∗^Cold ischaemic time (CIT) is defined as the time from donor aorta clamp to donor graft removal from ice prior to transplantation. S represents the standard deviation.

**Table 2:** Primary indications for transplant for adult patients who underwent liver transplantation in the study in 2015 and 2016 and the primary indications for transplant for adult patients from the cohort who were diagnosed with biopsy-proven T-cell mediated rejection (TCMR) within one year of transplantation.

Primary indication for transplant	*N* (total = 164)	Patients with TCMR *N* (total = 51)
Hepatitis C virus	39 (24%)	11 (21%)
Alcoholic cirrhosis	27 (16%)	11 (21%)
Hepatocellular carcinoma	17 (10%)	8 (16%)
Nonalcoholic fatty liver disease	17 (10%)	2 (4%)
Primary sclerosing cholangitis	15 (9%)	7 (14%)
Primary biliary cirrhosis	7 (4%)	1 (2%)
Hepatitis B virus	6 (4%)	0 (0%)
Autoimmune hepatitis	6 (4%)	2 (4%)
Subacute hepatic failure	6 (4%)	0 (0%)
Fulminant hepatic failure (drugs)	6 (4%)	7 (14%)
Others^∗^	18 (11%)	2 (4%)

^∗^Extrahepatic biliary atresia, alpha-1-antitrypsin deficiency, polycystic liver disease, Wilson's disease, cryptogenic cirrhosis, haemangioendothelioma, hyperoxaluria type 1, hepatitis D virus.

**Table 3:** Biopsy characteristics and histopathological outcome of the cohort.

Number of transplant recipients who underwent at least 1 biopsy in 12 months post-transplant	60 (37%)
Total number of biopsies	90
Biopsies/patient	Number of patients
1	41 (69%)
2	13 (21%)
3	3 (4.9%)
4	1 (1.6%)
5	2 (3.3%)
Time between LT and biopsy procedure	
Days	Number of biopsies
1–90	54 (60%)
91–180	16 (18%)
181–365	20 (22%)
Histopathological outcome of biopsies	Number of biopsies
T cell-mediated rejection (TCMR)	64 (71.1%)
Nonspecific changes	8 (8.9%)
Preservation-reperfusion injury	4 (4.4%)
Cholestatic hepatitis	4 (4.4%)
Chronic rejection	3 (3.3%)
Hepatitis C virus	3 (3.3%)
Other^∗^	4 (4.4%)

^∗^Steatosis, submassive necrosis, insufficient tissue, suboptimal biopsy.

**Table 4:** The biopsies with T cell-mediated rejection (TCMR) according to the Banff working group's consensus for global assessment categories and the rejection activity index (RAI).

Total RAI score						
Global assessment		1–2	3–5	6–7	8–9	Total
	Indeterminate	3	0	0	0	3 (4%)
	Mild	1	25	0	0	26 (41%)
	Moderate	0	6	18	1	25 (39%)
	Severe	0	0	1	9	10 (16%)
	Total	4 (5%)	31 (49%)	19 (30%)	20 (16%)	64 (100%)

Total RAI score = score for portal inflammation + bile duct damage + venous endothelial inflammation.

## Data Availability

Data are available from the corresponding author on request within the parameters of the ethics approval.

## Abbreviations

ALT: Alanine aminotransferase
ALP: Alkaline phosphatase
ANLTU: Australian national liver transplant unit
BDD: Bile duct damage
CIT: Cold ischaemic time
GGT: Gamma-glutamyl transpeptidase
GA: Global assessment
IV: Intravenous
LT: Liver transplantation
PI: Portal inflammation
RAI: Rejection activity index
RPAH: Royal Prince Alfred Hospital
SLHD: Sydney local health district
TCMR: T-cell mediated rejection
VEI: Venous endothelial inflammation.

## Supplementary Materials

Supplementary Materials A table showing the 2016 Banff Grading criteria (global assessment) and quantitative scoring (rejection activity index) with key changes in wording from the 1997 Banff schema underlined.
