# Supplementary material for: Banff 2016 Global Assessment and Quantitative Scoring for T Cell-Mediated Liver Transplant Rejection are Interchangeable
Source: J Transplant. 2023 Mar 27;2023:3103335. doi: 10.1155/2023/3103335 (PMC10070025; doi:10.1155/2023/3103335)
Supplement: Supplementary Materials — A table showing the 2016 Banff Grading criteria (global assessment) and quantitative scoring (rejection activity index) with key changes in wording from the 1997 Banff schema underlined. [file 3103335.f1.docx]

**Supplementary Table 1.** 2016 Banff Grading criteria (global assessment) and Quantitative scoring (rejection activity index) with key changes in wording from the 1997 Banff schema underlined.

| **Grading criteria (global assessment):**  • Indeterminate: Portal and/or perivenular inflammatory infiltrate that is related to an alloreaction, but shows insufficient tissue damage  to meet criteria for a diagnosis of mild acute rejection,  • Mild: Rejection-type infiltrate in a minority of the triads or perivenular areas, that is generally mild, and mostly confined within the  portal spaces for portal-based rejection and an absence of confluent necrosis/hepatocyte dropout for those presenting with isolated  perivenular infiltrates.  • Moderate: Rejection-type infiltrate, expanding most or all of portal tracts and/or perivenular areas with confluent necrosis/hepatocyte  dropout limited to a minority of perivenular areas.  • Severe: As above for moderate, with spillover into periportal areas and/or moderate-to-severe perivenular inflammation that extends  into the hepatic parenchyma and is associated with perivenular hepatocyte necrosis involving a majority of perivenular areas. |
| --- |
| **Quantitative scoring (rejection activity index [RAI]):**  **Score Criteria**  Portal inflammation:  1 Mostly lymphocytic inflammation involving, but not noticeably expanding, a minority of the triads.  2 Expansion of most or all of the triads, by a mixed infiltrate containing lymphocytes with occasional blasts, neutrophils, and eosinophils. If eosinophils are conspicuous and accompanied by edema and microvascular endothelial cell hypertrophy is prominent, acute  antibody-mediated rejection (AMR) should be considered.  3 Marked expansion of most or all of the triads by a mixed infiltrate containing blasts and eosinophils with inflammatory spillover into  the periportal parenchyma  Bile duct inflammation damage:  1 A minority of the ducts are cuffed and infiltrated by inflammatory cells and show only mild reactive changes such as increased  nuclear:cytoplasmic ratio of the epithelial cells.  2 Most or all of the ducts infiltrated by inflammatory cells. More than an occasional duct shows degenerative changes such as nuclear  pleomorphism, disordered polarity, and cytoplasmic vacuolization of the epithelium.  3 As above for 2, with most or all of the ducts showing degenerative changes or focal luminal disruption  Venous endothelial inflammation:  1 Subendothelial lymphocytic infiltration involving some, but not a majority of the portal and/or hepatic venules  2 Subendothelial infiltration involving most or all of the portal and/or hepatic venules with or without confluent hepatocyte necrosis/dropout involving a minority of perivenular regions.  3 As above for 2, with moderate or severe perivenular inflammation that extends into the perivenular parenchyma and is associated  with perivenular hepatocyte necrosis involving a majority of perivenular regions. |
